# Effect of a quality improvement package for intrapartum and immediate newborn care on fresh stillbirth and neonatal mortality among preterm and low-birthweight babies in Kenya and Uganda: a cluster-randomised facility-based trial

**DOI:** 10.1016/S2214-109X(20)30232-1

**Published:** 2020-07-22

**Authors:** Dilys Walker, Phelgona Otieno, Elizabeth Butrick, Gertrude Namazzi, Kevin Achola, Rikita Merai, Christopher Otare, Paul Mubiri, Rakesh Ghosh, Nicole Santos, Lara Miller, Nancy L Sloan, Peter Waiswa, Preterm Birth Initiative Kenya and Uganda Implementation Research Collaborative

**Affiliations:** a Institute for Global Health Sciences, University of California San Francisco, San Francisco, CA, USA; b Department of Obstetrics, Gynecology, and Reproductive Sciences, University of California San Francisco, San Francisco, CA, USA; c Center for Clinical Research, Kenya Medical Research Institute, Nairobi, Kenya; d Maternal, Newborn and Child Health Centre of Excellence, School of Public Health, College of Health Sciences, Makerere University, Kampala, Uganda; e Global Health Department of Public Health Sciences, Karolinska Institutet, Stockholm, Sweden

## Abstract

**Background:**

Although gains in newborn survival have been achieved in many low-income and middle-income countries, reductions in stillbirth and neonatal mortality have been slow. Prematurity complications are a major driver of stillbirth and neonatal mortality. We aimed to assess the effect of a quality improvement package for intrapartum and immediate newborn care on stillbirth and preterm neonatal survival in Kenya and Uganda, where evidence-based practices are often underutilised.

**Methods:**

This unblinded cluster-randomised controlled trial was done in western Kenya and eastern Uganda at facilities that provide 24-h maternity care with at least 200 births per year. The study assessed outcomes of low-birthweight and preterm babies. Eligible facilities were pair-matched and randomly assigned (1:1) into either the intervention group or the control group. All facilities received maternity register data strengthening and a modified WHO Safe Childbirth Checklist; facilities in the intervention group additionally received provider mentoring using PRONTO simulation and team training as well as quality improvement collaboratives. Liveborn or fresh stillborn babies who weighed between 1000 g and 2500 g, or less than 3000 g with a recorded gestational age of less than 37 weeks, were included in the analysis. We abstracted data from maternity registers for maternal and birth outcomes. Follow-up was done by phone or in person to identify the status of the infant at 28 days. The primary outcome was fresh stillbirth and 28-day neonatal mortality. This trial is registered with ClinicalTrials.gov, NCT03112018.

**Findings:**

Between Oct 1, 2016, and April 30, 2019, 20 facilities were randomly assigned to either the intervention group (n=10) or the control group (n=10). Among 5343 eligible babies in these facilities, we assessed outcomes of 2938 newborn and fresh stillborn babies (1447 in the intervention and 1491 in the control group). 347 (23%) of 1491 infants in the control group were stillborn or died in the neonatal period compared with 221 (15%) of 1447 infants in the intervention group at 28 days (odds ratio 0·66, 95% CI 0·54–0·81). No harm or adverse effects were found.

**Interpretation:**

Fresh stillbirth and neonatal mortality among low-birthweight and preterm babies can be decreased using a package of interventions that reinforces evidence-based practices and invests in health system strengthening.

**Funding:**

Bill & Melinda Gates Foundation.

## Introduction

Preterm birth remains a major contributor to perinatal mortality^1^, ^2^ and accounts for 35% of the 2·5 million annual neonatal deaths globally.^3^, ^4^ Spontaneous preterm birth is estimated to contribute to 30% of the 2·6 million annual stillbirths since the factors leading to preterm birth are also associated with stillbirth.^2^, ^5^ Efforts to implement the Every Newborn Action Plan (ENAP)^6^ and to reduce neonatal mortality in accordance with the Sustainable Development Goals should increasingly address preterm birth.

Globally, data on preterm birth rates are restricted because the current gold standard for gestational age estimation, first trimester ultrasound, is often not available in many low-income and middle-income countries.^7^ 8·6% of livebirths in Kenya and 6·6% of livebirths in Uganda are estimated to be preterm, according to research studies and population-based surveys.^1^ An estimated 14% of liveborn babies have low birthweight in sub-Saharan Africa (11·5% in Kenya; data unavailable for Uganda).^8^ Many of these susceptible babies do not survive their first day of life.

Intervening during the intrapartum and immediate newborn periods provides the greatest opportunity to save the most lives.^7^, ^9^ ENAP encourages the use of evidence-based practices such as appropriate use of antenatal corticosteroids, stimulation and resuscitation, immediate newborn care including kangaroo mother care, and management of neonatal sepsis.^6^, ^10^ Although these evidence-based practices and low-technology interventions can avert many preterm deaths if routinely implemented, uptake is low because of factors such as health system bottlenecks, inadequate provider training, and overall low quality of care.^9^, ^11^ Furthermore, key to these reductions is implementation of these evidence-based practices together, rather than individually.

**Research in context**

**Evidence before this study**

In the 2014 *Lancet Every Newborn* Series, Zulfiqar Bhutta and colleagues estimated that facility-based interventions delivered during labour and birth could avert 41% of all neonatal deaths and 70% of all stillbirths in 75 high-burden countries. Care for small and sick newborn babies could avert an additional 30% of all neonatal deaths in these countries. Prematurity is the leading cause of neonatal mortality, and over 80% of preterm births occur in Asia and sub-Saharan Africa. Thus, to reduce neonatal mortality in these settings, addressing prematurity is essential. Several Cochrane reviews have pointed to the efficacy of existing evidence-based practices to improve outcomes of preterm or low-birthweight infants, such as kangaroo mother care (average risk ratio for reduced risk of mortality due to low birthweight was 0·60 among eight trials) and provision of antenatal corticosteroids (average risk ratio for reduced risk of perinatal death was 0·72 among 15 studies; average risk ratio for reduced risk of neonatal death was 0·69 among 22 studies). However, in many low-income and middle-income countries, uptake of such effective practices is inadequate. Increasing coverage and uptake can be achieved through various approaches, such as audit and feedback to improve professional practice (a Cochrane review found a 4·3% increase in health-care professional compliance among 49 studies) and simulation-based training to improve provider competency (according to a systematic review with 68 studies) and clinical practice (according to a systematic review with 51 studies). Although quality improvement collaboratives alone showed varied efficacies on health outcomes in low-income and middle-income countries, when coupled with training, provider practice outcomes and patient health outcomes were improved (according to a systematic review and meta-analysis with 29 studies). In a large study in India, the WHO Safe Childbirth Checklist had no effect on stillbirth or early neonatal death; however, adherence to essential birth practices was higher in the intervention than the control group (62% *vs* 44% at 12 months).

**Added value of this study**

Informed by the 2014 *Lancet Every Newborn* Series and the 2012 WHO Born Too Soon Global Action Report on Preterm Birth, the East Africa Preterm Birth Initiative set out to decrease the burden of preterm birth in selected geographies. Our cluster-randomised controlled trial examined the effect of a package of interventions to reinforce uptake of existing evidence-based practices targeting the intrapartum and immediate newborn period in Kenya and Uganda. All facilities received maternity register data strengthening and provision of a modified Safe Childbirth Checklist with additional emphasis on preterm labour and care for newborn babies. Intervention facilities received provider training and mentoring using the PRONTO simulation and team training approach, and participation in quality improvement collaboratives. We found significantly lower odds of combined fresh stillbirth and 28-day mortality among preterm and low-birthweight infants born in intervention facilities than in control facilities, and significant reductions in pre-discharge mortality, perinatal mortality, fresh stillbirth, and 28-day neonatal mortality.

**Implications of all the available evidence**

Our results show that a quality improvement package that works in a coordinated fashion to promote evidence-based practices during the intrapartum and immediate newborn care window, without additional investment in advanced neonatal care or technology, is an effective strategy to improve outcomes among preterm infants. The mutually reinforcing nature of implementing a package of interventions, rather than individual or parallel strategies, might improve effectiveness in reducing prematurity-related mortality in low-income and middle-income countries.

Informed by ENAP, the *Lancet Every Newborn* Series,^6^, ^9^ local stakeholder priorities, and previous research trials, the East Africa Preterm Birth Initiative (PTBi-EA) together with our in-country partners set out to decrease the burden of preterm birth in Kenya and Uganda. With the intent to benefit all babies and not only those born preterm, PTBi-EA designed a comprehensive intrapartum and newborn care intervention package with an emphasis on preterm birth. Each intervention component was implemented synergistically to promote behaviour change for improved quality of care.

The four component PTBi-EA package included: maternity register data strengthening, a locally modified WHO Safe Childbirth Checklist (mSCC) to enhance preterm birth identification and management, quality improvement collaboratives based on the Institute for Healthcare Improvement model,^12^ and an adapted PRONTO International obstetric and newborn simulation and team training curriculum modified for preterm birth. The first two components were introduced in all facilities whereas all four components were only introduced in intervention facilities. The trial was designed as an integrated approach to optimise care for the mother–baby dyad, influencing provider behaviour at key moments during triage, labour, birth, and the newborn period.^13^, ^14^

Previous research informed the selection of these intervention components. A 2012 Cochrane review of provider audit and feedback showed modest increases in practice performance, suggesting that this quality improvement strategy might improve adherence to data reporting standards.^15^ A large randomised cluster trial that implemented the WHO Safe Childbirth Checklist in intervention facilities in India through an 8-month coaching programme showed no differences in stillbirth or early neonatal mortality between the intervention and control groups, though increases in evidence-based practices were observed.^16^ A systematic review of simulation-based training, including the PRONTO model, found strong evidence for improved clinical practice and some evidence for improved outcomes.^14^ PRONTO training focusing on intrapartum and immediate neonatal care improved use of evidence-based practices and decreased neonatal mortality in India and Mexico.^17^, ^18^ Lastly, quality improvement collaboratives alone do not affect patient outcomes consistently, but are effective in concert with provider training.^19^

The PTBi-EA intervention package was intended to reinforce existing data systems to improve data use, strengthen provider skills and team communication, and catalyse system improvement. We hypothesised that these interventions, implemented together as a package, could reduce the combined rate of fresh stillbirth and neonatal mortality among preterm and low-birthweight infants by 30% in intervention facilities compared with control facilities. This Article reports the trial's primary and secondary outcome findings; implementation and process outcomes will be described elsewhere.

## Methods

### Study design

An unblinded, pair-matched cluster-randomised controlled trial was implemented in public sector facilities in Uganda and public sector facilities in Kenya.^20^ All interventions were delivered at the facility level. The study was done in the Busoga region in eastern Uganda (with a population of 3 million) and in Migori County in western Kenya (with a population of 1 million).^21^ The regional Busoga and national neonatal mortality rates are similar (27 deaths per 1000 livebirths *vs* 28 deaths per 1000 livebirths, respectively), as are the stillbirth rates (17 stillbirths per 1000 pregnancies *vs* 16 stillbirths per 1000 pregnancies, respectively).^22^ The regional Migori County mortality rates are 19 deaths per 1000 livebirths and the national neonatal mortality rates are 22 deaths per 1000 livebirths; the regional Migori County stillbirth rates are 9·8 stillbirths per 1000 pregnancies and the national stillbirth rates are 13·2 stillbirths per 1000 pregnancies.^23^ The total fertility rate is 5·3 children per woman in Kenya and 6·1 children per woman in Uganda, with 24·3% of women in Kenya and 20·7% of women in Uganda having begun childbearing before the age of 19 years.^22^, ^23^ Women seeking care at government facilities in both regions predominantly live below the global poverty level.

The trial gained ethical approval from the Kenya Medical Research Institute, Makerere University School of Public Health, and the University of California, San Francisco Institutional Review Boards. Mothers of eligible babies who were alive at discharge provided informed consent for 28-day follow-up. The trial is registered on ClinicalTrials.gov, NCT03112018.

### Clusters

23 rural and peri-urban facilities were assessed as potential clusters. Inclusion criteria were 24-h labour and delivery services, at least 200 births per year, and having a comparable facility for pairing in the same country. Tertiary referral facilities were excluded. One county referral hospital in Kenya, and one district referral and one regional referral hospital in Uganda were assessed but not included in the study matching or randomisation. These three hospitals did not have comparable hospitals with which they could be paired.

The study clusters in Uganda were four district facilities (two public and two non-profit missionary facilities); all four facilities did caesarean sections and had a newborn care unit without capacity for continuous positive airway pressure and without an onsite paediatrician. Together, they had approximately 9000 deliveries per year (about 6% of the region's births) between 2016 and 2018.^21^ In Kenya, the 16 study clusters included 14 public and two non-profit missionary facilities; only two did caesarean sections, none had functional designated newborn care units when the study began, and none had an onsite paediatrician. One control site added caesarean section capacity while the study was ongoing. The 16 Kenya study facilities had approximately 11 000 deliveries per year between 2016 and 2018, representing 23% of the county's annual births.^24^

The study facilities in Uganda had between 1081 and 3142 deliveries per year with an average of two-to-three midwives covering each shift. The facilities in Kenya were smaller than those in Uganda, with a range of 310–1599 deliveries per year and an average of one-to-two midwives per shift. Deliveries were attended by one midwife with additional help called for when needed. The study did not add any additional clinical providers to the study sites during the course of the study. Intra-facility and inter-facility nursing staff rotations occur at regular intervals, approximately every 6 months in Kenya and approximately every 2 years in Uganda. First trimester ultrasound for pregnancy dating was not available at any of the 20 sites.

### Randomisation and matching

Using indicators collected from maternity registers covering a 1-year pre-intervention period (June 1, 2015, to May 31, 2016), we applied a non-bipartite matching algorithm to match ten facility pairs on the basis of country, monthly deliveries, deliveries to staff ratio, stillbirth rate, low-birthweight rate, and pre-discharge neonatal death rate (appendix p 1). We also assessed facility readiness at the time of matching. The resultant pairs were reviewed with field teams and five of the ten pairs were re-matched on the basis of local knowledge of functional level and facility type. After pair matching was finalised, a study statistician based in the USA randomly assigned one of each pair to the intervention group using R software. No allocation concealment was possible given the nature of the intervention.

### Intervention

**Figure 1:**
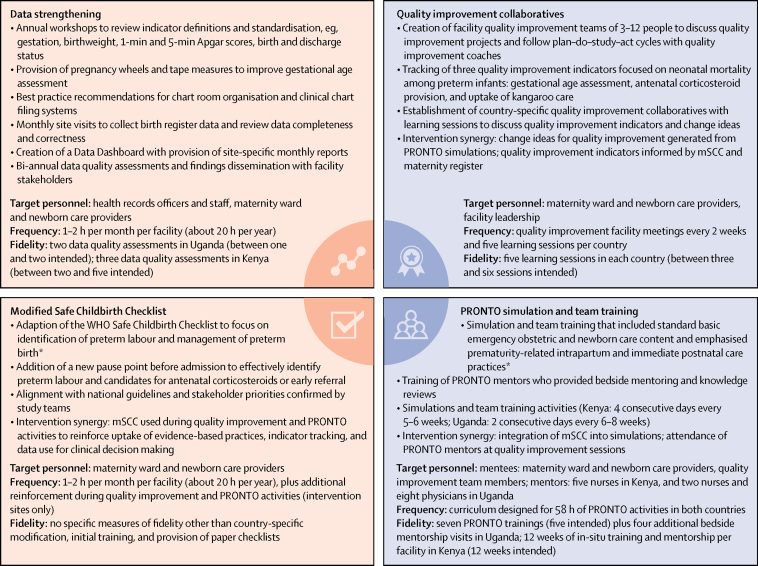
The four component intervention package mSCC=modified WHO Safe Childbirth Checklist. *Including accurate gestational age assessment, use of magnesium sulphate and antenatal corticosteroids, immediate skin to skin and breastfeeding, newborn resuscitation, and preterm feeding guidelines.

Figure 1 describes the intervention elements, frequencies, and fidelities. The package aimed to strengthen provider skills and teamwork, emphasising uptake of evidence-based practices, which include but are not limited to the use of antenatal corticosteroids, immediate skin to skin, breastfeeding, newborn resuscitation, and preterm feeding. It also reinforced data use for clinical and administrative decision making, with a focus on data quality for accurate gestational age assessment and key quality improvement indicator tracking.

Before study initiation, sites were documented to have basic equipment and supplies, such as functional digital scales and neonatal bag valve masks for preterm and term newborn babies. If unavailable, PTBi-EA provided these supplies to standardise resource availability across all study sites, whereby these expenses did not exceed US$50 000 in either country. Introduction of data strengthening and the mSCC to all 20 clusters began before Sept 30, 2016, after which PRONTO and quality improvement collaboratives were added to the ten intervention facilities.

The study obtained a waiver of consent to collect de-identified maternity register data. Birth outcome data were captured for all deliveries listed in maternity registers between Oct 1, 2016, and May 31, 2018, in Uganda and between Oct 1, 2016, and April 30, 2019, in Kenya. Pre-intervention data were also collected between March 1 and Sept 30, 2016, in Uganda, and between June 1 and Sept 30, 2016, in Kenya. In Kenya, the study was extended to compensate for a 5-month nurses' strike and a 2-month doctors' strike that greatly reduced delivery volume. The study team abstracted deidentified maternity register data on a monthly basis on all births to obtain overall denominators for facility rates. We excluded register entries for mothers admitted for antenatal complications or referred without delivery (ie, threatened preterm or false labour) and babies born outside the facility.

### Participants and data collection

Mothers with babies born alive weighing less than 2500 g or between 2500 g and 2999 g with a recorded gestational age of less than 37 weeks were approached to consent for follow-up to 28 days. In addition to capturing all babies with low birthweight, most of whom are either preterm or small for gestational age,^25^ we also estimated that the upper limit of 3000 g would capture 90–97% of infants younger than 34 weeks and 60–70% of infants younger than 37 weeks.^20^ We used this definition because measuring birthweight in addition to gestational age has greater reliability than estimating gestational age alone. Fresh stillborn babies meeting the same eligibility criteria were also included.^26^ Stillborn and liveborn babies weighing less than 1000 g were excluded from our primary analysis because they are considered previable in both countries, but were evaluated in secondary analyses. Infants not meeting these criteria or listed in the registers as abortions, macerated stillbirth, or without entries for birthweight and gestational age were excluded.

Mothers of eligible live infants were approached for consent to be contacted by phone for 28-day follow-up. Trained facility providers (Kenya and Uganda) or community health volunteers (Kenya) obtained consent before the mother was discharged home. Providers or community health volunteers reviewed the maternity register daily to ascertain eligible deliveries. If the mother left the facility before she was able to consent, the study team used contact information provided in the facility records to reach her by telephone or in person through community health volunteers. Women agreeing to participate provided either written consent (Kenya and Uganda) or verbal consent if followed up retrospectively by telephone (Uganda). In Kenya, an additional 7-day follow-up call was made. Study staff made three attempts to reach a mother by phone before sending a study nurse (Uganda) or a community health volunteer (Kenya) to trace the mother at her home. Eligible stillbirths and pre-discharge deaths were included in analyses from register data, while mothers were not consented for the 28-day follow-up.

Study staff entered data into an encrypted Open Data Kit database on tablets or laptops. Follow-up data were collected using paper forms, which was then delivered to each country's central study office where the data were entered into an encrypted Django web-based database. Each mother and each infant was assigned unique separate identifiers that were linked. Unique identifiers were used to link 28-day outcomes with register data. The electronic data were maintained on secure systems with access limited to the study principal investigators, epidemiologists, and designated study staff.

### Outcomes

The primary outcome was the combined incidence of fresh stillbirth and 28-day neonatal mortality among eligible babies weighing between 1000 g and 2500 g, or between 2500 g and 2999 g with a recorded gestational age of less than 37 weeks. After publication of our protocol, we reworded our primary outcome to clarify that babies weighing 3000 g or above with a recorded gestational age of 37 weeks would be excluded. Babies under these cutoffs are included. Secondary outcomes included perinatal mortality (fresh stillbirth plus 7-day mortality), facility-based maternal mortality (captured in the maternity register), pre-discharge mortality (death of a liveborn baby before facility discharge, noted in the discharge status), and neonatal mortality for babies born alive and weighing less than 1000 g.

Additional post-hoc analyses included caesarean section rates, individual components of the primary outcome (eg, fresh stillbirth and neonatal mortality), and fresh stillbirth and pre-discharge mortality for all registered births.

### Statistical analysis

As described in the protocol,^20^ with an estimated sample size of 4000 eligible births, an assumed intraclass correlation coefficient of 0·03, and a baseline incidence of fresh stillbirth plus neonatal mortality among eligible infants of 25%, the study would have 80% power at the 5% significance level to detect a 30% relative reduction in the primary outcome. We assumed that the intervention and control groups were balanced with respect to delivery volume. No effect sizes were hypothesised about secondary outcomes and the sample size was not adjusted for multiple comparisons.

During the extended health worker strikes in Kenya, we reassessed our sample size. We accounted for the higher-than-expected birth volumes in Uganda, the need for a longer-than-expected data collection period in Kenya, data from the pre-intervention period estimate on primary outcomes, and a post-hoc one-tailed test on the primary outcome. Given a type I error of 0·05, power of 80%, a one-tailed test, and a balanced (1:1 for control and intervention group) sample, a sample size of 1133 preterm births was required per study group. The sample size was increased by 35% (to 1530 preterm births per study group) to account for clustering and loss to follow-up or missing information.

Range and logic checks were applied to the maternity register and follow-up data using the mySQL data management and development software. Out-of-range data and data with discrepancies in eligibility or critical outcomes were sent to designated field staff to review and resolve. Biologically implausible or invalid data were recoded as missing before analysis.

χ^2^ and t tests were used to compare the study groups by sociodemographic, reproductive health, and facility characteristics. An intention-to-treat analysis was done using logistic regression and generalised estimating equations with robust variance to account for clustering of births within facilities and to adjust for pairing of facilities. All primary and secondary outcomes, except neonatal mortality among babies weighing less than 1000 g, were assessed in the intention-to-treat population. Liveborn babies whose birthweight did not meet inclusion criteria (< 1000 g) were examined as a separate population for the secondary outcome only (because they fall outside viability definitions in Kenya and Uganda). We used an exchangeable correlation structure. At the facility level, we also estimated the difference in log odds of the outcomes within each matched pair and did a paired t-test on the average difference, weighted by the delivery volume.

Although the study started with relatively balanced groups, there were only ten matched pairs, and adjustment for potential confounding factors was not possible at the facility level because the number of observations (n=10) was too small. Therefore, we did an individual-level analysis in which the unit of analysis was a delivery. We used a directed acyclic graph to identify potential confounders and mediators on which data were available (appendix pp 6–7). The main results were adjusted for pairing and clustering only. To examine robustness of the main results, we additionally adjusted for potential confounders and examined the effect of mediators. We also did sensitivity analyses to examine if the key results changed because of changes in conditions (eg, initiation of caesarean section at a single facility mid-study, but its paired facility did not have this capacity) or if any individual matched pair disproportionately influenced the overall results. Analogous analyses were done to assess secondary outcomes. Significance tests were two-tailed at the 5% level. To show that the results were not affected by multiple comparison, we corrected the p values using Bonferroni corrections. Analyses were done using SPSS version 23 and STATA version 15 (StataCorp). The trial is registered with ClinicalTrials.gov, NCT03112018.

### Role of the funding source

The funder of the study reviewed the study design, but had no role in data collection, data analysis, data interpretation, or writing of the report. National and community advisory boards provided input on intervention priorities. Health facility providers, managers, and local authorities were involved in implementation activities and influenced the focus and content of those activities on the basis of their roles and priorities. DW, PO, EB, CO, PM, RG, NLS, and PW had full access to all the data in the study. The corresponding author had final responsibility for the decision to submit for publication.

## Results

Of the 23 public sector facilities initially assessed, 20 were included in the trial (four facilities in Uganda and 16 facilities in Kenya). Ten facilities were randomly assigned to the intervention group and ten to the control group. We achieved fidelity to the expected number of trainings for the intervention components (figure 1). Despite the pair-matched random assignment, the control facilities had higher annual mean delivery volumes (1139 deliveries [SD 826]) than the intervention facilities (901 deliveries [605]). Baseline median caesarean rates among facilities that offered caesarean section were 20% (IQR 16–20) in the control group and 47% (11–47) in the intervention group (appendix p 1). One control facility began doing caesarean sections during the course of the trial. All facilities had 85% average completion rates of maternity register data, including gestational age, birthweight, Apgar scores, and birth outcomes. At the time of matching, the median facility readiness score was 71% (IQR 62–76) in the control group and 67% (61–72) in the intervention group. At the end of the study, the median scores were 61% (53–78) and 56% (44–71; p=0·54).

**Figure 2:**
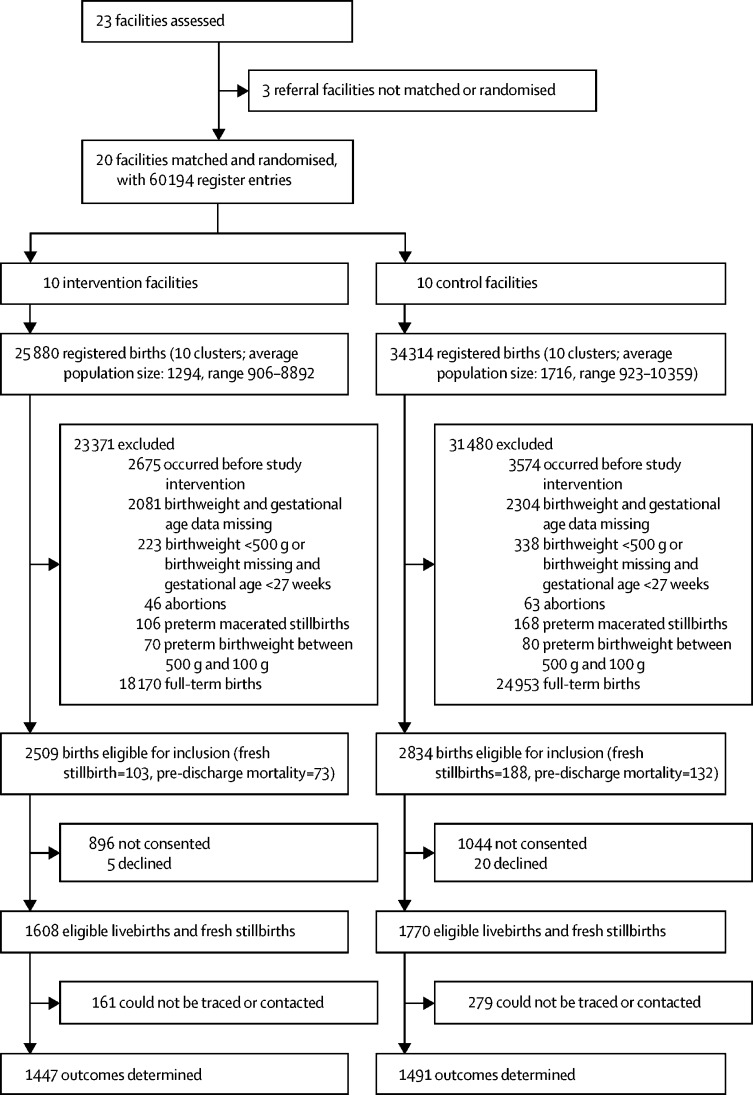
Trial profile

Maternity register data on 60 194 women were abstracted between Oct 1, 2016, and May 31, 2018, in Uganda, and from Oct 1, 2016, to April 30, 2019, in Kenya (figure 2). Data on 54 851 records were excluded because they were full-term births, the births occurred before the study intervention, birthweight and gestational age data were missing, birthweight was less than 500 g, gestational age was less than 27 weeks, or they were abortions or macerated stillbirths. Thus, the trial is limited to eligible livebirths or stillbirths. 5343 births were eligible for follow-up; however, 25 mothers declined and 1940 were not approached for consent. Among the 3378 babies to be followed up, 291 were fresh stillborn babies, 205 were newborn babies who died before facility discharge, and 440 were infants who were lost to 28-day follow-up. 1491 control and 1447 intervention group participants (total n=2938) had complete primary outcome data. Similar enrolment and follow-up trends were observed between the study groups.

Fewer women were younger than 18 years old and more women had caesarean deliveries in the control group than in the intervention group (table 1). Infant characteristics were similar between the groups, including low birthweight and preterm birth (recorded gestational age less than 37 weeks) rates. More eligible deliveries were multiple gestation in the control group (382 [22%] of 1770) than in the intervention group (313 [20%] of 1608). Infants born in the control group (225 [16%] of 1447) had more Apgar scores lower than 7 at 5 min after birth than those born in the intervention group (112 [7%] of 1512).

**Table 1:** Maternal and newborn characteristics for eligible births*

		**Control group**	**Intervention group**
**Maternal characteristics**			
Maternal age, years			
	<18	155/1385 (11·2%)	200/1288 (15·5%)
	18–35	1157/1385 (83·5%)	1033/1288 (80·2%)
	>35	73/1385 (5·3%)	55/1288 (4·3%)
Caesarean delivery		311/1359 (22·9%)	172/1289 (13·3%)
Multiple gestation		382/1770 (21·6%)	313/1608 (19·5%)
**Infant characteristics**			
Low birthweight (<2500 g)		1343/1770 (75·9%)	1187/1608 (73·8%)
Gestational age <37 weeks		1182/1725 (68·5%)	1098/1577 (69·6%)
Apgar score <7 at 5 min after birth for livebirths†		225/1447 (15·5%)	112/1512 (7·4%)
Sex			
	Male	840/1755 (47·9%)	758/1603 (47·3%)
	Female	915/1755 (52.1%)	845/1603 (52·7%)

Data are n/N (%), where n is the numerator for the specific category in the control or intervention group, and N is the total number of non-missing observations for the respective variable in the control or intervention group.

* Eligible births are fresh stillbirths and livebirths of babies weighing between 1000 g and 2499 g irrespective of gestational age, or between 2500 g and 2999 g with a recorded gestational age less than 37 weeks.

† Excluding stillbirths.

For the primary outcome, 347 (23%) of 1491 infants in the control group were stillborn or died in the neonatal period compared with 221 (15%) of 1447 infants in the intervention group (adjusted odds ratio 0·66 (95% CI 0·54–0·81), p<0·0001; table 2). Perinatal mortality and pre-discharge newborn mortality were also higher among newborn babies in the control group than in the intervention group (table 2). Mortality among babies born alive between 500 g and 999 g was high (about 80%) in both groups. Fresh stillbirth and neonatal mortality were higher in babies in the control group than in those in the intervention group. In the seven facilities with caesarean capability, more mothers had caesarean sections in the control facilities (311 of 883, 35·2%) than in the intervention facilities (172 of 643, 26·8%), and more mothers had caesarean sections during the study (355 of 1854, 19·1%) than before the study (217 of 839, 25·9%).

**Table 2:** Effect of the intervention on the primary, secondary, and additional outcomes among eligible births* or among livebirths weighing less than 1000 g

	**Control group**	**Intervention group**	**Adjusted odds ratio (95% CI)**†	**p value**
**Primary outcome**				
Fresh stillbirth and neonatal death (combined)	347/1491 (23·3%)	221/1447 (15·3%)	0·66 (0·54–0·81)	<0·0001
**Secondary outcomes**				
Perinatal mortality: fresh stillbirth + 7-day mortality	312/1485 (21·0%)	199/1447 (13·8%)	0·67 (0·56–0·81)	<0·0001
Pre-discharge newborn mortality	132/1542 (8·6%)	73/1439 (5·1%)	0·57 (0·48–0·68)	<0·0001
Pre-discharge maternal mortality	12/1441 (0·8%)	6/1359 (0·4%)	‡	..
Neonatal mortality among newborn babies weighing <1000 g	20/25 (80·0%)	26/31 (83·9%)	‡	..
**Additional outcomes**				
Preterm fresh stillbirth	188/1770 (10·6%)	103/1608 (6·4%)	0·69 (0·57–0·83)	<0·0001
Preterm neonatal mortality	159/1303 (12·2%)	118/1345 (8·8%)	0·72 (0·58–0·90)	0·004
Caesarean delivery (at all facilities)	311/1359 (22.9%)	172/1289 (13.3%)	0·63 (0·38–1·04)	0·072
Caesarean delivery (at caesarean-capable facilities)§	311/883 (35·2%)	172/643 (26·8%)	0·67 (0·41–1·12)	0·12

For pre-discharge mortality, register data were used for eligible infants. Data are n/N (%), were n is the number with outcomes in the control or intervention group and N is the total number of non-missing eligible births in the control or intervention group.

* Eligible births are fresh stillbirths and livebirths of babies weighing between 1000 g and 2499 g irrespective of gestational age, or between 2500 g and 2999 g with a recorded gestational age less than 37 weeks.

† Odds ratios accounted for matching of facilities and clustering of births within facilities.

‡ The numbers are too small for the models to converge to provide stable results.

§ Among births in facilities with capability for caesarean section.

The odds ratio (OR; OR 0·70, 95% CI 0·49–0·99) of the primary outcome at the facility level within each pair is presented in the appendix (p 2). In the individual analysis, after accounting for matching and clustering, the intervention was associated with lower odds (OR 0·66, 95% CI 0·54–0·81) of being a fresh stillbirth or neonatal death among eligible infants than the control (table 2). Additional analyses controlling for caesarean delivery, multiple gestation, infant sex, country of intervention, birthweight, delivery volume, and facility readiness did not change the estimate. Adjustment for Apgar score at 5 min after birth changed the effect (OR 0·84, 95% CI 0·60–1·18). The estimated intraclass correlation coefficient for the primary outcome was 0·0196. Results remain unchanged after correcting for multiple comparisons (results not shown).

In secondary and additional analyses adjusted for matching and clustering (table 2), the intervention was associated with lower odds of perinatal mortality (OR 0·67, 95% CI 0·56–0·81) and pre-discharge mortality (OR 0·57, 95% CI 0·48–0·68) than the control. Lower odds of fresh stillbirth (OR 0·69, 95% CI 0·57–0·83) and neonatal mortality (OR 0·72, 95% CI 0·58–0·90) was observed in the intervention than in the control group. The intervention was not associated with a significant change in the proportion of women receiving caesarean section. Pre-discharge maternal mortality (18 observations) and mortality among infants born alive weighing less than 1000 g (56 observations) had too few observations to do cluster-adjusted analyses.

We explored the effect of our intervention package on all births occurring in the facilities for fresh stillbirth and pre-discharge newborn mortality only; results are in the appendix (p 3). No unintended or harmful effects were identified.

## Discussion

Preterm birth and intrapartum complications account for nearly 70% of neonatal mortality worldwide^4^ and the factors leading to preterm birth are also causally associated with stillbirth.^5^ This trial found that, above and beyond the efforts of data strengthening and introduction of the mSCC in all study facilities, an intrapartum quality of care improvement package including PRONTO simulation and team training as well as quality improvement collaboratives had a large significant effect on fresh stillbirth and neonatal mortality among low-birthweight and preterm babies. The trial was not powered to identify independent effects among fresh stillbirth, pre-discharge newborn mortality, and neonatal mortality. Regardless, the intervention package also had a large effect on fresh stillbirth, pre-discharge mortality, and neonatal mortality among low-birthweight and preterm babies.

When we adjusted for 5-min-after-birth Apgar scores, the magnitude of the intervention effect was attenuated from an OR of 0·66 to 0·84. Because an Apgar score reflects the wellbeing of an infant immediately after birth, we believe that it is a potential mediator on the causal pathway and is a reflection of increased use of evidence-based practices during the intrapartum and immediate newborn period in intervention facilities. However, further analyses are warranted.

Our trial found a strong effect for fresh stillbirth and early (7-day) newborn mortality (OR 0·67, 95% CI 0·56–0·81). These results are consistent with other trials in low-income and middle-income countries that used an integrated package approach. For example, a Nepal study assessing an intervention that included quality improvement and provider training found a significant decrease in intrapartum mortality (ie, stillbirth or infant death within the first 24 h) and a non-significant decrease in early neonatal mortality.^27^ Another Ugandan study that focused on workforce training, provision of equipment and supplies, and strengthening of health information systems showed a non-significant decline in pre-discharge neonatal mortality from 17% to 9%.^28^

We believe our intervention was effective in large part because the package, which spanned labour, delivery, and immediate newborn periods, worked synergistically to increase the awareness and consistent use of practices for preterm infants known to improve outcomes. We selected a locally relevant package of strategies that targeted provider skills, knowledge, and experience, and that provided a mechanism to identify and overcome weaknesses and bottlenecks for providing essential care.^13^ Furthermore, the practical approach of working with the existing strengths and limitations in real health systems suggests that this intervention is scalable in Kenya, Uganda, and other similar settings without significant investment in neonatal care technologies. Cost-effectiveness analyses are underway.

Despite the increased focus on preterm survival, caesarean rates were lower in the intervention than control facilities among those offering caesarean, and increased in control facilities from baseline. This difference was not due to the one control facility that added caesarean capacity, as it did few caesarean deliveries during enrolment. Rather, two control sites probably did more caesarean deliveries because one received more referrals thanks to staffing and resource availability and the second because of its location in the main town. Although higher caesarean rates in control facilities might indicate higher-risk births and could translate to worse outcomes, these births presumably also occurred in the presence of higher-level care. The result for the primary outcome did not change after adjustment for caesarean section.

The trial used robust statistical methods that controlled for matching and clustering of births in facilities, thereby addressing some of the study limitations. The strike in Kenya reduced delivery volume and also resulted in some staff attrition and reassignment to different divisions or facilities. Whereas Uganda exceeded its projected sample sizes by cluster and completed data collection early, data collection was extended in Kenya to achieve a re-estimated sample size in alignment with available funding. Although we did not achieve the required sample size as stated a priori, the intraclass correlation coefficient that was smaller than the a priori assumption and the effect size that was higher than the a priori assumption adequately powered the study.

Furthermore, because of our practical approach in which we used existing health facility staff or community health volunteers to initially screen and consent participants for follow-up, we were unable to consent approximately a third of eligible babies. Those babies who were excluded from the study were not different from those included with respect to pre-discharge neonatal mortality or maternal mortality, maternal age, caesarean section, multiple gestation, infant sex, or gestational age (appendix pp 4–5). A higher proportion of low-birthweight infants were included in the study sample rather than excluded; however, when comparing intervention and control groups, the difference was small and statistically non-significant, suggesting selection bias is unlikely although it cannot be ruled out. Regardless of these limitations, the trial's robust cluster-adjusted analyses were able to detect significant effects on the primary and various secondary outcomes.

We aimed to improve data quality in control and intervention groups to enhance attention to newborn care and preterm birth; however, the extent to which relying on maternity register data for identification of eligible newborn babies affected data quality, as compared with a parallel data collection system, is unknown. Our approach was similar to the Better Birth trial,^16^ which assessed outcomes from facility registers followed by contacting caregivers after hospital discharge. Nonetheless, investment in existing data systems is essential for long-term capacity development and offers a unique platform for evaluation of quality improvement-focused projects, as shown by a facility-based quality improvement initiative in Ghana that relied on the government's health information system to assess outcomes.^29^

Although the accuracy of gestational estimation is questionable, and the use of birthweight probably misclassifies some newborn babies who are small for gestational age as preterm, our eligibility criteria enabled us to capture the most fragile babies. These eligibility criteria were a novel approach to defining preterm birth solely on the basis of birthweight and gestational age data that were provider-documented and routinely recorded. Use of both birthweight and gestational age variables guided by INTERGROWTH-21st standards helped address data quality concerns.^30^ Our primary outcome included fresh stillbirth because of the high percentage of stillbirths that are also preterm and the possibility of early neonatal death being misclassified as stillbirth, as suggested by the Born Too Soon Action Group.^26^ Although providers' accuracy in classifying livebirths and fresh and macerated stillbirths could have improved because of data strengthening efforts, some inaccuracy continued to exist, as we reclassified a small number of stillbirths with 1-min Apgar scores above zero as livebirths. To our knowledge, we are the first to use this approach and recommend it as a practical strategy when using maternity register data and the introduction of early ultrasound for gestational age assessment is not feasible.

In summary, a synergistic intrapartum and immediate newborn package with a focus on preterm birth had a large significant effect on reducing fresh stillbirth and newborn mortality among preterm and low-birthweight babies. These promising results merit future replication in similar and distinct settings to address the pervasive problem of preterm birth. Additionally, further inquiry is needed to find out whether the improvements in mortality are sustained through the first year of life and whether there are subsequent effects on post-neonatal morbidity.

## Data sharing

We will share deidentified individual participant-level data underlying the primary results in this Article, as well as relevant metadata, study protocol, and analysis plan. Data will be available to researchers who present ethically sound proposals to the corresponding author, beginning 6 months after publication until 3 years after publication date of this Article. Requestors will need to sign a data access agreement that is subject to approval by the study principal investigators. After 3 years the data will be uploaded to the UCSF's data sharing site and will be freely available but without any assistance beyond the metadata files.
