# Supplementary material for: Effect of a quality improvement package for intrapartum and immediate newborn care on fresh stillbirth and neonatal mortality among preterm and low-birthweight babies in Kenya and Uganda: a cluster-randomised facility-based trial
Source: Lancet Glob Health. 2020 Jul 22;8(8):e1061–70. doi: 10.1016/S2214-109X(20)30232-1 (PMC7388203; doi:10.1016/S2214-109X(20)30232-1)
Supplement: Supplementary appendix [file mmc1.pdf]

THE LANCET
Global Health

Supplementary appendix

This appendix formed part of the original submission and has been peer reviewed. We post it as supplied by the authors.

Supplement to: Walker D, Otieno P, Butrick E, et al. Effect of a quality improvement package for intrapartum and immediate newborn care on fresh stillbirth and neonatal mortality among preterm and low-birthweight babies in Kenya and Uganda: a cluster-randomised facility-based trial. *Lancet Glob Health* 2020; **8:** e1061–70.

Appendix

Table 1: Facility characteristics at matching

	Control N=10 facilities	Intervention N=10 facilities	Total N=20 facilities	p value^
Monthly delivery volume, mean (SD)	93 (82)	75 (51)	84 (67)	0·57
Deliveries to staff ratio, mean (SD)	63 (28)	52 (25)	58 (26)	0·39
Stillbirth proportion, median % (IQR)	1 (0·3-4)	2 (1-4)	1 (1-4)	0·38
LBW proportion, median % (IQR)	4 (3-7)	6 (2-6)	5 (3-6)	0·97
Pre-discharge newborn mortality, median % (IQR)	0 (0-1)	0.5 (0-1)	0 (0-1)	0·83
Caesarean section rate at 6 caesarean-capable facilities, median % (IQR)	20 (16-20)	47 (11-47)	21 (18-27)	<0.001

^Two tailed paired t-test for means, Mann-Whitney U-test for medians

Table 2: Difference in the primary outcomes for facility pairs

Pair	Intervention (odds)*	Control (odds)*	Odds Ratio~
1	0·03	0·09	0·32
2	0·24	0·28	0·85
3	0·36	0·35	1·02
4	0·15	0·16	0·91
5	0·10	0·15	0·67
6	0·62	0·71	0·88
7	0·60	0·38	1·56
8	0·17	0·14	1·21
9	0·07	0·37	0·20
10	0·07	0·13	0·50
Exponentiated mean log odds ratio (95% CI):			0·70 (0·49-0·99)
			p value=0·04^

*weighted by the number of deliveries within each facility
^two tailed paired t-test
~ Intervention log odds - control log odds = log odds ratio (exponentiated)

Table 3: Additional outcomes among all births occurring at study facilities with adjustment for matching and clustering

	Control		Intervention		Odds Ratio	95% CI		p value
	n_c/N_c	%	n_i/N_i	%		Lower	Upper	
Fresh stillbirth	674/30726	2·2	300/23194	1·3	0·84	0·59	1·19	0·32
Pre-discharge newborn mortality	363/30726	1·2	217/23194	0·9	0·90	0·74	1·09	0·29

$n_{c/i}$ = number with outcomes in the control/intervention arm $N_{c/i}$= Total number of non-missing eligible births in the control/intervention arm

Table 4a and 4b. Characteristics of the infants who were excluded from the study versus those who were included, aggregated totals (4a) and separated by intervention and control (4b).

4a.

Characteristics	n_1 #	Eligible but excluded*	n_2 #	Eligible and included	p-value!
Preterm fresh stillbirth	2436	0 (0)	2909	9·2 (268)	NA
Pre-discharge mortality	2436	4·0 (97)	2909	3·7 (108)	0·61
Pre-discharge maternal mortality	2196	0·3 (7)	2772	0·7 (18)	0·10
Apgar at 5 min <7 live births	2436	4·8 (118)	2909	9·9 (287)	<0·001
Maternal age (years)	2423		2895		
<18		10·7 (259)		11·4 (329)	
18-35		83·5 (2024)		83·5 (2417)	
>35		5·8 (140)		5·2 (149)	0·47
Caesarean delivery (mothers)	2404	23·0 (552)	2863	21·6 (617)	0·22
Multiple gestation	2436	19·6 (477)	2909	21·1 (614)	0·17
Low birth weight (<2500 g)	2436	64·8 (1579)	2909	75·8 (2206)	<0·001
Sex, male	2436	46·2 (1125)	2909	47·7 (1387)	0·28
Birth weight in grams (Mean, SD)	2436	2252 (451)	2909	2174 (440)	<0·001
Gestational age in weeks (Mean, SD)	2268	35·1 (3·4)	2848	35·0 (3·5)	0·051

*Exclusion due to failure to consent, declined participation, could not trace/contact

#n_1 and n_2 are total denominators for the control and intervention group, respectively.

!Chi-squared test of proportions or unpaired t-test for means.

4b.

	Eligible but excluded*					Eligible and included				
	n_1 #	Control = 1361	n_2 #	Intervention = 1075	p-value!	n_1 #	Control = 1474	n_2 #	Intervention = 1435	p-value!
Preterm fresh stillbirth	1361	0 (0)	1075	0 (0)	NA	1474	11·9 (175)	1435	6·5 (93)	<0·001
Pre-discharge mortality	1361	5·2 (71)	1075	2·4 (26)	<0·001	1474	4·1 (61)	1435	3·3 (47)	0·22
Pre-discharge maternal mortality	1229	0·2 (3)	967	0·4 (4)	0·48	1425	0·8 (12)	1347	0·5 (6)	0·19
Apgar at 5 min <7 live births	1361	5·4 (74)	1075	4·1 (44)	<0·001	1474	12·7 (187)	1435	7·0 (100)	<0·001
Maternal age (years)	1354		1069			1468		1427		
<18		9·3 (126)		12·4 (133)			9·6 (141)		13·2 (188)	
18-35		83·8 (1134)		83·3 (890)			84·9 (1246)		82·1 (1171)	
>35		6·9 (94)		4·3 (46)	0·002		5·5 (81)		4·8 (68)	0·008
Caesarean delivery (mothers)	1336	26·5 (354)	1068	18·5 (198)	<0·001	1433	26·9 (385)	1430	16·2 (232)	<0·001
Multiple gestation	1361	22·6 (308)	1075	15·7 (169)	<0·001	1474	21·9 (322)	1435	20·4 (292)	0·32
Low birth weight (<2500 g)	1361	66·8 (909)	1075	62·3 (670)	0·02	1474	76·9 (1133)	1435	74·8 (1073)	0·19
Sex, male	1361	45·6 (620)	1075	47·0 (505)	0·49		47·8 (705)		47·5 (682)	0·87
Birth weight in grams (Mean, SD)	1361	2229 (456)	1075	2283 (443)	0·004	1474	2144 (446)	1435	2206 (431)	<0·001
Gestational age in weeks (Mean, SD)	1274	35·1 (3·3)	994	35·1 (3·4)	0·95	1437	34·9 (3·5)	1411	35·0 (3·4)	0·44

*Exclusion due to failure to consent, declined participation, could not trace/contact

#n_1 and n_2 are total denominators for the control and intervention group, respectively.

!Chi-squared test of proportions or unpaired t-test for means.

Figure 1. Conceptual diagram showing the relationship between the intervention and the outcome along with the potential confounders and mediators.

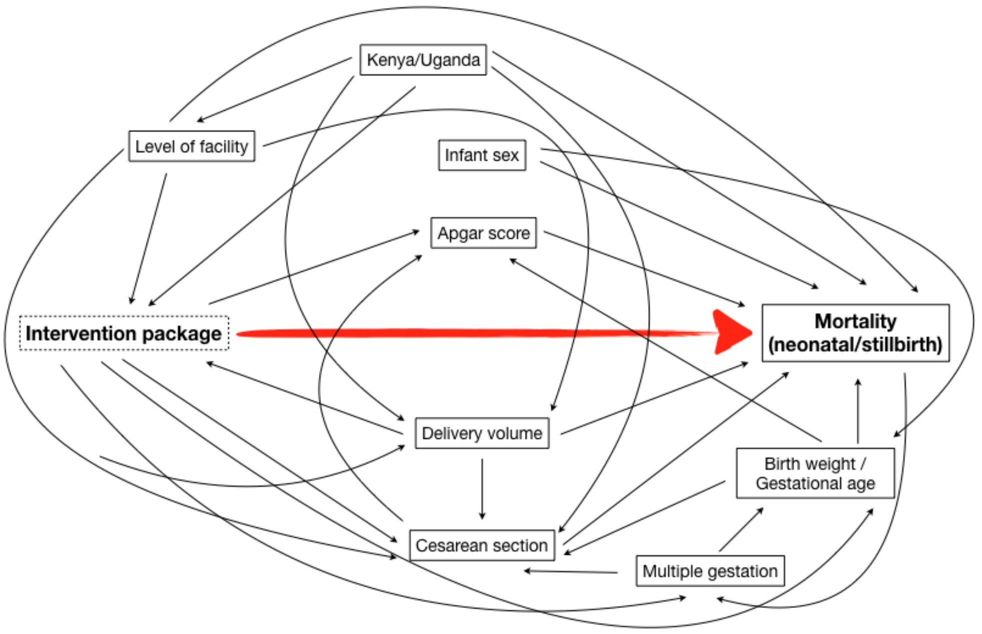

We used this directed acyclic graph to show the relationship under investigation within the context of potential confounders and mediators.

- The red arrow shows the hypothesized relationship under investigation.
- **Delivery volume** depends on the level of facility. A higher-level facility that has high delivery load is more likely to have more c-sections, it is more likely to have higher volume of poor outcomes because they get more serious and referred cases. Because of the higher volume there may be greater investment in improving facility resources and/or capacity building of human resources. In contrast, because of the high work load they may have less time to invest in the simulation training or participate in QI collaboratives.
- **Apgar score** is a potential mediator because it indicates health status of newborn immediately after birth and it is also likely to have been influenced by the intervention, thus on the causal pathway.
- Performance of **cesarean section** may be influenced by the intervention package. Providers may be more or less likely to turn to a cesarean section if preterm birth is suspected. Similarly, increased vigilance of intrapartum care may result in higher incidence of cesarean section for intrauterine fetal distress. Performance of cesarean section may impact the likelihood of a very preterm infant being delivered by CS only to die shortly after birth vs becoming a fresh stillbirth.

- **Infant sex.** Male infants are at higher risk of mortality, in general, and we also know that female infants are at higher risk of born with intrauterine growth retardation in low- and middle-income countries. There may be provider or societal biases that lead to greater efforts to save infants of one sex or the other.
- Although the intervention was the same, its implementation is likely to have differences because of inherent differences between the sites in **Kenya and Uganda**. The facilities in these countries are different in terms of the services they provide and the number of deliveries they cater to. There may also be differences in the underlying provider skill or competencies for managing preterm labor and birth.
- **Birthweight** together with gestational age are the largest determinants of newborn survival. Among preterm infants there is a wide array of birthweights and gestational ages that are directly related to newborn survival.

CONSORT 2010 checklist

Section/Topic	Item No	Standard Checklist item	Extension for cluster designs
**Title and abstract**			
	1a	Identification as a randomised trial in the title	Identification as a cluster randomised trial in the title
	1b	Structured summary of trial design, methods, results, and conclusions (for specific guidance see CONSORT for abstracts)[i,ii]	See table 2
**Introduction**			
**Background and objectives**	2a	Scientific background and explanation of rationale	Rationale for using a cluster design
	2b	Specific objectives or hypotheses	Whether objectives pertain to the cluster level, the individual participant level or both
**Methods**			
**Trial design**	3a	Description of trial design (such as parallel, factorial) including allocation ratio	Definition of cluster and description of how the design features apply to the clusters
	3b	Important changes to methods after trial commencement (such as eligibility criteria), with reasons	
**Participants**	4a	Eligibility criteria for participants	Eligibility criteria for clusters
	4b	Settings and locations where the data were collected	
**Interventions**	5	The interventions for each group with sufficient details to allow replication, including how and when they were actually administered	Whether interventions pertain to the cluster level, the individual participant level or both
**Outcomes**	6a	Completely defined pre-specified primary and secondary outcome measures, including how and when they were assessed	Whether outcome measures pertain to the cluster level, the individual participant level or both
	6b	Any changes to trial outcomes after the trial commenced, with reasons	
**Sample size**	7a	How sample size was determined	Method of calculation, number of clusters(s) (and whether equal or unequal cluster sizes are assumed), cluster size, a coefficient of intracluster correlation (ICC or *k*), and an indication of its uncertainty
	7b	When applicable, explanation of any interim analyses and stopping guidelines	

**Randomisation:**			
**Sequence generation**	8a	Method used to generate the random allocation sequence	
	8b	Type of randomisation; details of any restriction (such as blocking and block size)	Details of stratification or matching if used
**Allocation concealment mechanism**	9	Mechanism used to implement the random allocation sequence (such as sequentially numbered containers), describing any steps taken to conceal the sequence until interventions were assigned	Specification that allocation was based on clusters rather than individuals and whether allocation concealment (if any) was at the cluster level, the individual participant level or both
**Implementation**	10	Who generated the random allocation sequence, who enrolled participants, and who assigned participants to interventions	Replace by 10a, 10b and 10c
	10a		Who generated the random allocation sequence, who enrolled clusters, and who assigned clusters to interventions
	10b		Mechanism by which individual participants were included in clusters for the purposes of the trial (such as complete enumeration, random sampling)
	10c		From whom consent was sought (representatives of the cluster, or individual cluster members, or both), and whether consent was sought before or after randomisation
**Blinding**	11a	If done, who was blinded after assignment to interventions (for example, participants, care providers, those assessing outcomes) and how	
	11b	If relevant, description of the similarity of interventions	
**Statistical methods**	12a	Statistical methods used to compare groups for primary and secondary outcomes	How clustering was taken into account
	12b	Methods for additional analyses, such as subgroup analyses and adjusted analyses	
**Results**			
**Participant flow (a diagram is strongly recommended)**	13a	For each group, the numbers of participants who were randomly assigned, received	For each group, the numbers of clusters that were randomly assigned, received intended

		intended treatment, and were analysed for the primary outcome	treatment, and were analysed for the primary outcome
	13b	For each group, losses and exclusions after randomisation, together with reasons	For each group, losses and exclusions for both clusters and individual cluster members
**Recruitment**	14a	Dates defining the periods of recruitment and follow-up	
	14b	Why the trial ended or was stopped	
**Baseline data**	15	A table showing baseline demographic and clinical characteristics for each group	Baseline characteristics for the individual and cluster levels as applicable for each group
**Numbers analysed**	16	For each group, number of participants (denominator) included in each analysis and whether the analysis was by original assigned groups	For each group, number of clusters included in each analysis
**Outcomes and estimation**	17a	For each primary and secondary outcome, results for each group, and the estimated effect size and its precision (such as 95% confidence interval)	Results at the individual or cluster level as applicable and a coefficient of intracluster correlation (ICC or k) for each primary outcome
	17b	For binary outcomes, presentation of both absolute and relative effect sizes is recommended	
**Ancillary analyses**	18	Results of any other analyses performed, including subgroup analyses and adjusted analyses, distinguishing pre-specified from exploratory	
**Harms**	19	All important harms or unintended effects in each group (for specific guidance see CONSORT for harms[iii])	
**Discussion**			
**Limitations**	20	Trial limitations, addressing sources of potential bias, imprecision, and, if relevant, multiplicity of analyses	
**Generalisability**	21	Generalisability (external validity, applicability) of the trial findings	Generalisability to clusters and/or individual participants (as relevant)
**Interpretation**	22	Interpretation consistent with results, balancing benefits and harms, and considering other relevant evidence	
**Other information**			

**Registration**	23	Registration number and name of trial registry
**Protocol**	24	Where the full trial protocol can be accessed, if available
**Funding**	25	Sources of funding and other support (such as supply of drugs), role of funders

** Note: page numbers optional depending on journal requirements*

[i] Hopewell S, Clarke M, Moher D, Wager E, Middleton P, Altman DG, et al. CONSORT for reporting randomised trials in journal and conference abstracts. *Lancet* 2008, 371:281-283

[ii] Hopewell S, Clarke M, Moher D, Wager E, Middleton P, Altman DG at al (2008) CONSORT for reporting randomized controlled trials in journal and conference abstracts: explanation and elaboration. *PLoS Med* 5(1): e20

[iii] Ioannidis JP, Evans SJ, Gotzsche PC, O'Neill RT, Altman DG, Schulz K, Moher D. Better reporting of harms in randomized trials: an extension of the CONSORT statement. *Ann Intern Med* 2004; 141(10):781-788.
